# The Antiviral Properties of Cyclosporine. Focus on Coronavirus, Hepatitis C Virus, Influenza Virus, and Human Immunodeficiency Virus Infections

**DOI:** 10.3390/biology9080192

**Published:** 2020-07-28

**Authors:** Paulina Glowacka, Lidia Rudnicka, Olga Warszawik-Hendzel, Mariusz Sikora, Mohamad Goldust, Patrycja Gajda, Anna Stochmal, Leszek Blicharz, Adriana Rakowska, Malgorzata Olszewska

**Affiliations:** 1 Department of Dermatology, Medical University of Warsaw, 02-008 Warsaw, Poland; paulinaglowacka6@gmail.com (P.G.); olga.warszawik@wp.pl (O.W.-H.); drmariuszsikora@gmail.com (M.S.); gajda.patrycja.pg@gmail.com (P.G.); stochmal.anna@gmail.com (A.S.); lblicharz@gmail.com (L.B.); adriana.rakowska@wum.edu.pl (A.R.); malgorzata.olszewska@wum.edu.pl (M.O.); 2 Department of Dermatology, G. Marconi University of Rome, 00193 Rome, Italy; drmgjgoldust@gmail.com; 3 Department of Dermatology, University Medical Center Mainz, 55131 Mainz, Germany; 4 Department of Dermatology, University Hospital Basel, 4031 Basel, Switzerland

**Keywords:** calcineurin inhibitors, cyclosporine, tacrolimus, hepatitis flu, AIDS, coronavirus, human papilloma virus infection, human herpesvirus, infections, cyclophilin

## Abstract

This review updates current knowledge regarding the risk of viral infections, including COVID-19, in patients treated with cyclosporine. We also shortly refer to bacterial infections and parasitic infestations in patients treated with cyclosporin. Cyclosporine is an immunosuppressive drug, which is widely used in medicine, including in the treatment of autoimmune skin diseases in dermatology, rheumatology, ophthalmology and nephrology, and in organ transplantation. A usual concern associated with immunosuppressive treatment is the potential risk of infections. Interestingly, several data indicate a relatively low risk of infections, especially viral infections, in patients receiving cyclosporine. It was shown that cyclosporine exerts an inhibitory effect on the replication of some viruses, or may have a potentially beneficial effect on the disease course in infections. These include hepatitis C, influenza virus, rotavirus, human immunodeficiency virus and coronavirus infections. Available data indicate that cyclosporine may have a beneficial effect on COVID-19, which is caused by the coronavirus SARS-COV2.

## 1. Introduction

Cyclosporine is a calcineurin inhibitor that acts selectively on T cells. It was isolated from *Tolypocladium inflatum* fungi in 1970, and now it is widely used as an immunosuppressive drug [1,2] in such areas of medicine as dermatology, transplantology, nephrology, rheumatology and ophthalmology. The main approved indications are psoriasis, atopic dermatitis, the prevention of graft rejection following solid organ transplantation and bone marrow transplantation, the treatment of graft-versus-host disease, endogenous uveitis, Behçet uveitis, nephrotic syndrome and rheumatoid arthritis [3].

An increased risk of infections is believed to be an adverse effect of cyclosporine. However, some authors have indicated that patients receiving cyclosporine are at a low risk of common infections [1,2], with the risk being comparable to that in individuals taking the placebo [4]. During the COVID-19 pandemic, concerns are being raised about the safety of patients treated with cyclosporine. It has been debated whether cyclosporine may have an effect on infection with the SARS-CoV2 (severe acute respiratory syndrome coronavirus 2), which causes COVID-19, and on the disease course [5].

This article reviews the literature related to the influence of cyclosporine on the occurrence of viral, bacterial and parasitic infections. Data published from 2010 to March 2020 were collected by searching the Pubmed, EBSCO and Scopus databases, using the terms “cyclosporine” (and its spelling variations) or “calcineurin inhibitors” and “virus”, “bacteria” and “parasite” (and their variations), including specific names of infectious diseases. Searches also were performed to identify articles related to “COVID-19”, “SARS-CoV2” and “coronavirus”.

## 2. Cyclosporine and Cyclophilins

**Figure 1:**
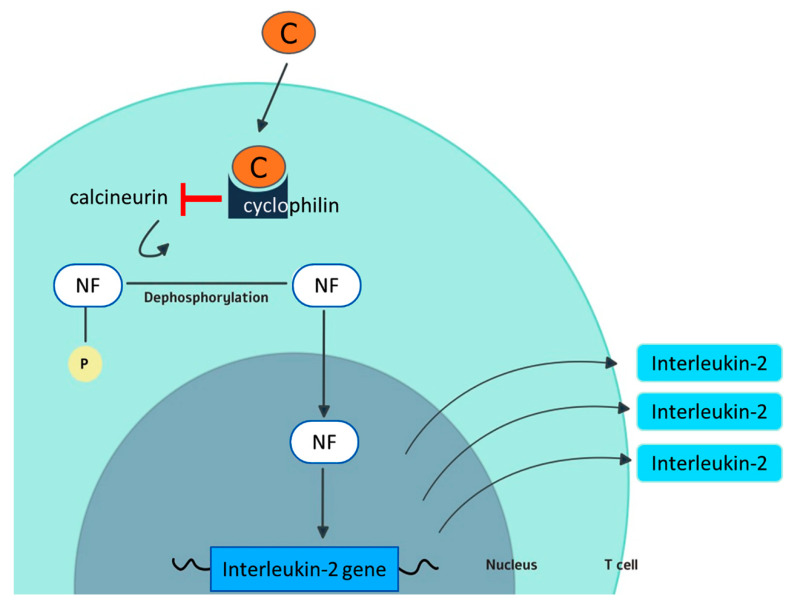
Mechanism of action of cyclosporine. In the cytoplasm of T cells, cyclosporine (C) forms a complex with cyclophilin. The cyclosporine–cyclophilin complex interferes with the phosphatase activity of the enzyme calcineurin. As a consequence, calcineurin cannot dephosporylate the nuclear factor (NF) that limits IL-2 production by T cells and its full activation. Cyclophilin is also believed to be one of the targets for the antiviral activity of cyclosporine.

Cyclosporine exerts its activity through binding with cyclophilin A (Figure 1).

Cycophilins are a ubiquitously distributed protein group, belonging to the immunophilin family [6]. Cyclophilin A is a key pathogenic player in numerous inflammatory diseases. It also is a mediator for many cardiovascular diseases, and a crucial mediator in Alzheimer’s disease and amyotrophic lateral sclerosis [7]. The expression of cyclophilin increases during inflammatory diseases, such as psoriasis or rheumatoid arthritis [8].

Cyclophilin A facilitates the replication of several viruses and is considered a potential target for antiviral therapy [6,8]. One of the main goals of current pharmacology is the development of cyclosporine-like molecules, which inhibit cyclophilin A, without exerting immunosuppressive activity [6].

## 3. Cyclosporine and Viral Infections

A cross-sectional and longitudinal study on the prevalence of past and reactivated viral infections in patients with psoriatic arthritis treated with cyclosporine brought unexpected results [8]. Cyclosporin was not associated with an increased risk of viral infections. Out of a group of 238 consecutive patients diagnosed within eight years from baseline, 225 patients were included in the analysis. In total, 177 and 174 patients were assessed in the 6-month and 12-month follow-up visits, respectively. The screening for viral infections included: hepatitis B virus (HBV), hepatitis C virus (HCV), herpes simplex virus 1–2 (HSV 1-2), herpes zoster virus (HZV), human herpesvirus 6 (HHV-6), Epstein–Barr virus (EBV), human immunodeficiency virus 1–2 (HIV 1-2), cytomegalovirus (CMV) and parvovirus B19. During the follow-up no reactivation or new-onset viral infection was observed [9]. Based on these findings and the available data in the literature, the authors concluded that cyclosporine in monotherapy or in combination with other immunosuppressants is not associated with increased risk of viral infections, and that it may be the treatment of choice in HCV-positive patients, who require immunosuppressive therapy. 

### 3.1. Hepatitis B Virus

The basis for the latest research into cyclosporine and the hepatitis B virus (HBV) is a number of studies conducted before 2010 [9,10]. They indicated the inhibiting role of cyclosporine on HBV replication, in a dose-dependent manner in vitro. Cyclosporine interfered with calcium signaling in the cytoplasm and limited the replication of HBV. The decreased expression of HbsAg and HBeAg, and the promotion of HbcAg entry into the hepatic nucleus, were also described with concomitant cyclosporine treatment [10]. 

Different results were obtained later, in an HBV-transfected mouse model study [11]. A high replication of HBV in mice treated with cyclosporine, which decreased after the withdrawal, was demonstrated. The authors concluded that HBV persistence was caused by the impaired immune function of CD8 T-cells [11].

The identification of sodium taurocholate co-transporting polypeptide (NTCP) as a functional HBV receptor expressed in hepatocytes allows for the further investigation of cyclosporine’s capacity to interfere with HBV infection [12]. 

Human hepatocyte cultures were infected with HBV while escalating cyclosporine concentration [12]. The blockage of HBV entry into hepatocytes was reported. The inhibition of NTCP hepatic transporter activity was highlighted as a method of the restriction of HBV infection, and was independent of the effect on cyclophilin and calcineurin. This observation during the study, and the distinct lack of an effect on HBV infectivity in the case of cyclophilin silencing, prompted researchers to exclude classic cyclosporine pathways as a mechanism of the inhibition of HBV replication. 

Reports also indicated that sensitivity to cyclosporine presented at former phases of the viral life cycle—before or during virus inoculation, but not when HBV cellular replication had already started. Thus, cyclosporine hardly affected the cells which had already been transfected with HBV [12].

The results of another cell culture study were published almost simultaneously, and present similar conclusions [13]. The inhibition of HBV entry into hepatocyte culture in a dose-dependent manner was observed. Moreover, the lack of correlation between antiviral activity and binding to cyclophilin and calcineurin was noted. Furthermore, cyclosporine analogs, which were also tested during the study, showed a higher inhibiting potency than cyclosporine [13].

In contrast, some reports considering treatment options for patients with HBV and HCV infection indicated that the use of cyclosporine was associated with a moderate (1–10%) risk of HBV reactivation during psoriasis treatment, including the need for concomitant anti-HBV prophylactic therapy and the checking of HBV DNA levels every three months [14].

According to the Medical Board of the National Psoriasis Foundation, all patients with psoriasis who are candidates for cyclosporine should undergo screening for hepatitis B virus infection, using triple serology testing: hepatitis B surface antigen, hepatitis B surface antibody and hepatitis B core antibody [10]. We consider this a good approach, until more research data are available.

### 3.2. Hepatitis C Virus

HCV replication depends on the interaction between cyclophilin B and protein 5B. Cyclosporine A inhibits their binding, thereby limiting viral replication [6].

A case report of a 14-year-old patient with severe atopic dermatitis and concomitant HCV infection was presented in the literature [15]. The patient underwent 12 months of systemic low-dose cyclosporine treatment, and another 12 months of pulsed cyclosporine treatment. The analysis of HCV RNA level showed a significant reduction in HCV RNA during the first phase of low-dose cyclosporine therapy [15].

Another study indicated the important role of nonstructural protein 5A (NS5A), which binds to HCV RNA in HCV-infected cells [16]. This complex is sensitive to cyclophilin inhibitors. Calcineurin inhibitors, like cyclosporine, disrupted HCV RNA biding by NS5A at the same time as decreasing the production of HCV particles in vitro [16].

A case study described a 48-year-old patient with severe chronic plaque psoriasis and HCV infection [17]. The treatment lasted 38 months and consisted of five cycles of cyclosporine (5 mg/kg/day), with the duration of one cycle ranging from 3 to 6 months. The levels of aminotransferases and HCV RNA were measured and demonstrated a reduction without complete normalization during 38 months of cyclosporine therapy [17].

Another case of a 75-year-old female was reported [18]. The patient had suffered from pustular psoriasis and HCV infection with liver function deterioration for 1 year. After eight weeks of cyclosporine treatment, the level of liver enzymes decreased in comparison with laboratory test results on admission [18].

Some reports demonstrated that cyclosporine therapy might inhibit the expression of RNA and proteins of HCV in vitro [19]. Moreover, the introduction of a cyclosporine pre-treatment onto hepatic cell cultures revealed the desirable inhibition of HCV. The necessity of continuous cyclosporine treatment was highlighted because of research showing a rebound viral status after the withdrawal of cyclosporine from HCV-infected cell cultures [19].

In conclusion, these data speak clearly in favor of the anti-HCV activity of cyclosporine, with some authors indicating even that cyclosporine may provide a valuable treatment solution in some patients with hepatitis C.

### 3.3. Hepatitis D Virus

The Hepatitis D virus (HDV) is an RNA virus that takes advantage of the HBV envelope proteins to infect the hepatic cells [12]. Sodium taurocholate co-transporting polypeptide (NTCP) is the HDV receptor that plays a role in the infection of hepatocytes. Data indicate that HDV entry via sodium taurocholate co-transporting polypeptide may be directly inhibited by cyclosporin A in cell cultures. It was achieved independently from the cyclophilin pathway that was mentioned earlier as a potential antiviral point of reference for cyclosporine [12]. These data pointing to the possible beneficial effect of cyclosporine on HDV infection have to be interpreted in the context of animal model data from many years ago, which indicated that treatment with cyclosporine may increase viraemia, and that upon discontinuation of treatment, HDV-RNA levels either return to pretreatment levels or became negative. In view of these conflicting results, further studies are needed to justify a more clear conclusion [20].

### 3.4. Hepatitis E Virus

Hepatitis E virus (HEV) infection affects numerous recipients of organ transplants, causing the development of chronic hepatitis [21]. Chronic HEV infection is generally associated with immunosuppressive treatment. However, data on immunosuppression and HEV infection are scarce. Subgenomic and infectious models displayed cyclosporine as a promoting factor in HEV replication, in a dose-dependent manner. It has been shown that cyclophilins A and B inhibit the replication of HEV. This, in the opinion of the authors of these studies, might explain the ability of cyclosporin to promote HEV infection [21].

### 3.5. Cytomegalovirus

A study conducted in 23 CMV-seropositive heart and heart–lung transplant recipients and 7 healthy controls showed that cyclosporine-induced immunosuppression resulted in a marked change of T-cell response, with reduction of T-cell polyfunctionality [22]. This led the authors to the hypothesis that immunosuppression with cyclosporine reduces polyfunctionality, which may result in increased infection risk in this patient group [22].

Another study, performed in 95 heart–lung transplants recipients, showed that 45.1% of the patients treated with cyclosporine had at least one symptomatic or asymptomatic CMV infection [23]. The respective number in patients treated with tacrolimus was 15.9% (*p* = 0.002). The authors indicated that cyclosporine therapy is an independent risk factor of CMV infection [23].

In 2017, the first case report was published to describe a non-HIV-infected patient who developed CMV anterior uveitis after 3 months of the topical use of cyclosporine 0.05% ophthalmic emulsion [24]. Conversely, another study provided evidence that cyclophilin A (CypA) was associated with H2O2-mediated CMV replication. As a consequence, targeting CypA with such substances as cyclosporine was considered a potential therapeutic option in HCV infection [25]. In vitro data indicate that cyclosporine, but not tacrolimus, inhibits cytomegalovirus infection via a cyclophilin-dependent pathway. 

Thus, studies into the effect of cyclosporine on CMV infection provide conflicting results, but the only big clinical study may indicate a tendency towards the increased risk of CMV infection in patients treated with cyclosporine. 

### 3.6. Herpes Simplex Virus

A study was conducted to compare the frequency of herpes keratitis after penetrating keratoplasty in two groups of patients [26]. A total of 88 subjects receiving cyclosporine treatment, in comparison with 185 subjects without cyclosporine therapy, were evaluated for the adverse effects of immunosuppressive treatment. The results showed that herpes keratitis occurred in 31.8% of the patients receiving cyclosporine, compared to 16.8% in the control group (*p*  =  0.023), indicating the possible promoting role of cyclosporine in HSV infection [26]. Contradicting results were obtained in several recent studies, which showed the beneficial effect of topical cyclosporine 2% eye drops in the treatment of herpetic stromal keratitis.

Furthermore, case reports provide conflicting conclusions [27]. A case report presented data on a 49-year-old man on cyclosporine treatment, after liver transplantation, with the exacerbation of seizures. Chronic encephalitis with HSV-1 etiology was identified after 2 months of cyclosporine therapy. The acute phase of encephalitis was asymptomatic, which may imply the role of cyclosporine in the development of HSV-1 infection [27]. Another study reported herpes simplex esophagitis in a renal transplant patient treated with cyclosporine. The beneficial effect of cyclosporine on HSV-induced erythema multiforme has also been documented in case reports [28]. 

The very limited data on the effect of cyclosporine on HSV infection provide conflicting results, with some very vague indications of possible beneficial effect.

### 3.7. Human Papilloma Virus

The risk factors of cutaneous viral infection during immunosuppressive treatment were investigated in 486 kidney transplant patients [29]. A total of 187 subjects had infectious skin lesions, with the predominance of infection warts of human papilloma virus etiology. The development of viral warts was promoted by cyclosporine treatment [29]. Human papilloma virus (HPV) replication increase, due to cyclosporine therapy, was also confirmed in a group of 69 kidney recipients with pathological changes in the oral cavity related to human papilloma virus. The results were based on histopathological examination and PCR tests [30].

### 3.8. Influenza Virus

Several studies confirmed the inhibiting activity of cyclosporine against the influenza virus in a dose-dependent manner in vitro. The inhibition was independent of cyclophilin A blockage, and cyclosporine did not affect the viral polymerase [31,32]. In one of the studies, cyclosporine was added at various phases of virus replication, which revealed that cyclosporine therapy did not disrupt the adsorption, internalization or RNA replication of the influenza virus in culture cells. The inhibiting effect of cyclosporine was disclosed after viral protein synthesis [32].

Cyclosporine H, which differs from cyclosporine by the substitution of the L-methyl valine at position 11 with its D-isomer, has even been suggested as beneficial in the treatment of Influenza virus flu [31].

### 3.9. Rotavirus

An increasing titer of cyclosporine was used in rotavirus-infected mouse models. The resolution of diarrhea and improvement of clinical measurements after cyclosporine therapy led to the conclusion that cyclosporine presented antiviral features against rotavirus [33,34]. It was also demonstrated that cyclosporine treatment may retrieve type I IFN production and improve immunity against rotavirus [34]. The study on the mouse model infected with rotavirus showed another advantage, i.e., diminished recovery time after diarrhea onset observed in stool test and the histopathology sample of the small intestine of the evaluated subjects [34]. The authors of this study formulated the hypothesis that cyclosporine may develop into an anti-Rotavirus drug.

### 3.10. Herpes Zoster Virus

The risk of herpes zoster during psoriasis treatment was assessed in a cohort study published in 2015 [35]. The study included 95,941 patients with psoriasis, and 522,616 person-years of follow-up were collected from the database. Statistical analysis indicated the lack of association between systemic cyclosporine treatment and herpes zoster virus infection [35]. However, some case reports from transplantology indicate the possibility of severe herpes zoster virus infections, especially herpes zoster meningitis, in patients treated with cyclosporine [36]. 

### 3.11. Human Immunodeficiency Virus

Reports published in 2010 failed to document any benefit achieved by adding cyclosporine to antiretroviral therapy, as management of the early stage of HIV infection in 45 evaluated patients [37]. Despite expectations, the study revealed no significant changes in the quantity of proviral DNA or CD4+ T-cells, and discredited the effectiveness of cyclosporine in acute HIV-1 infection [37]. In 2017, researchers returned to the topic of concomitant cyclosporine and antiretroviral therapy during HIV infection [38]. No improvement associated with immune recovery was observed, but the researchers extrapolated that some anti-integration effect of cyclosporine during the HIV life cycle was possible [38]. While searching for the cure for HIV infection, the researchers studied cyclosporine-mediated HIV-1 inhibition in vitro. The significant inhibiting effect of cyclosporine on HIV-infection was observed in cell cultures [39]. According to some authors, SUN1 protein limited the nuclear entry of HIV-1 DNA. The inhibition effect depended on cyclosporine and cellular cyclophilin A (CypA) interaction [40]. The latest reports tackled the issue of another protein, TRIM5α, engaging in HIV-1 infection. This molecule inhibited HIV with the following CypA-capsid lack of interaction. This is another issue which requires further research in order to explain the role of cyclosporine in HIV infection [41]. The question about its possible role in HIV infection remains open, with some evidence pointing to the possible inhibitory effect of cyclosporine on HIV replication.

#### 3.11.1. Human Herpesvirus-8

The human herpesvirus-8 (HHV-8), also called Kaposi sarcoma herpesvirus (KSHV), is known to be the causative factor for Kaposi sarcoma, especially in patients with AIDS [42]. A case of a 42-year-old patient with atopic dermatitis treated with cyclosporine was described. After 12 weeks of treatment, the patient developed new skin changes with lymphedema and inguinal lymphadenopathy on the legs. A biopsy revealed HHV-8 positive Kaposi sarcoma [42]. In 2016, a case of Kaposi sarcoma in the genital area was first documented in a kidney transplant recipient. The change of immunosuppressive treatment from cyclosporine to everolimus caused the resolution of Kaposi sarcoma within 1 year [43].

#### 3.11.2. Coronaviruses

**Figure 2:**
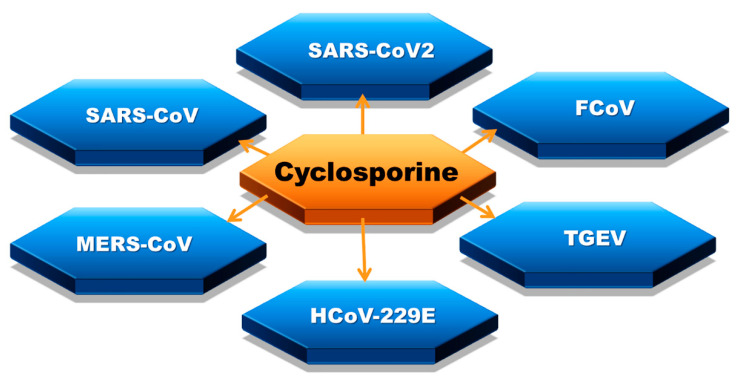
Coronaviruses affected by cyclosporine: severe acute respiratory syndrome coronavirus 2 (SARS-CoV2), severe acute respiratory syndrome coronavirus (SARS-CoV or SARS-CoV1), feline coronavirus (FCoV), Middle East respiratory syndrome coronavirus (MERS-CoV), transmissible gastroenteritis virus (TGEV), human coronavirus 229E (HCoV-229E).

Coronaviruses (CoV) mainly induce respiratory and gastrointestinal diseases in numerous mammalian species, including humans. Certain human CoVs are responsible for common cold symptoms, contrary to MERS and SARS, which also belong to the family of coronaviruses and are associated with high mortality [8]. The effect of cyclosporine therapy on coronavirus infection course has been widely studied (Figure 2). The topic became extremely prescient with the circumstances of the COVID-19 epidemic [44,45,46,47,48].

The inhibition of SARS replication by cyclosporine was proven in the cell culture model. Viral RNA and protein levels were almost undetectable after adding a non-toxic quantity of cyclosporine [49]. The same study was conducted on human coronavirus 229E, with the same conclusions obtained. One study demonstrated the contribution of cyclosporin-inhibited cyclophilin to the antiviral effect [50].

Another study showed that cyclosporine decreased SARS replication. However, the limitation of cyclophilin A in a cell had no effect on the replication [51].

An experiment with the method of high-throughput screening for the effect of cyclosporine on coronaviruses was conducted. The inhibition of replication and functional interaction between cyclophilin and viral proteins was observed. However, despite high cyclosporine concentration, the limitation of infection in cell cultures was not wholly obtained, and a certain part was not resistant to MERS infection [52]. In 2018, the reduction of MERS-CoV replication was proven in the human in vitro and ex vivo models [53].

## 4. Cyclosporine and Bacterial Diseases

Urinary tract infection is the most frequent bacterial infection after kidney transplantation [54]. It was shown that one of the significant risk factors of urinary tract infection is immunosuppressive therapy with cyclosporine [54]. A study on urinary tract infections caused by *Escherichia coli* in the renal graft was conducted. Cyclosporine weakened the expression of TLR4 and, as a consequence, distinctly decreased cytokine production and neutrophil migration, and increased bacterial levels [55]. Cyclosporine also interfered with Nod1 expression, which is responsible for the innate antibacterial defense in kidneys [56].

The evaluation of 4554 patients after kidney transplantation in a 10-year period was performed [57]. Tuberculosis was diagnosed in 2.4% of them. The risk factors for developing tuberculosis among renal transplant recipients were studied, and cyclosporine appeared to be one of them [57].

There are some case reports, which may point to the beneficial effect of cyclosporine in selected cases of bacterial diseases [58]. A case of a patient with staphylococcus-associated marginal keratitis was reported. The 0.05% ophthalmic emulsion of cyclosporine was administered with complete improvement in clinical examination [58].

## 5. Cyclosporine and Parasitic Diseases

Demodex is a common parasite presenting in low quantities on the human skin. A study including 45 patients was conducted. Out of this group, 15 cyclosporine-receiving patients underwent skin biopsy, and had their Demodex density measured at the beginning, and in the first and third months, of the treatment. Demodex density was statistically higher in comparison with the control group after one and three months of immunosuppressive therapy. Therefore, it may be concluded that cyclosporine treatment might be associated with a high number of mites and demodicosis [59].

A study concerning oxidative stress damage in *Trypanosoma cruzi* was conducted. Pre-incubation with cyclosporine revealed cyclosporine to be a preventative factor for oxidative stress damage in this parasite [60]. Conversely, *Trypanosoma cruzi* may neutralize antimicrobial peptide trialysin, which is inhibited by cyclosporine, as demonstrated by the authors [61].

A study conducted on a host–pathogen system with *Daphnia magna-Pasteuria ramosa* showed that cyclosporine might limit the disease resistance of an invertebrate organism to a parasite [62].

The influence of cyclosporine on *Schistosoma mansoni* was also examined. The incubation of parasites with cyclosporine revealed the malproduction, decreased number and retardation of egg development in *Schistosoma mansoni*. 

Researchers conducting studies on P-glycoprotein, which is an ATP-dependent transporter involved in the efflux out of cells, indicated that cyclosporine was a possible inhibitor of that transporter. As a consequence, cyclosporine cumulated in the parasitic tissue of *Echinococcus granulosus*, and was associated with the lower viability of the larval stage of the parasite [63].

A study conducted in 2011 provided the preliminary explanation of how cyclosporine might inhibit the development of *Plasmodium.* The antimalarial activity of cyclosporine might be explained by the phenomenon of the permeabilization and aggregation of the sphingomyelin-rich membrane network produced by the parasites during their development in erythrocytes [64].

Cyclosporine A was also demonstrated to exert a cytostatic and cytotoxic effect on *Leishmania donovani* in cell cultures. Cyclosporine acted on promastigotes and amastigotes via the blockage of calcineurin phosphatase and cyclophilin-dependent thermotolerance, respectively [65]. Conversely, other studies proved there was no significant inhibiting effect of cyclosporine on amastigotes, and the authors did not recommend cyclosporine as an anti-leishmanial drug [66].

## 6. Conclusions

In conclusion, the general belief that cyclosporine, through its immunosuppressive activity, has a purely negative impact on viruses and infections in humans finds no good confirmation in the data of the current literature. These data are very sparse, but tend to indicate that in some cases cyclosporine may exert antiviral activity (Table 1). It has to be considered that all data are very preliminary, and in a vast majority of cases insufficient for drawing conclusions regarding the clinical practice.

The possible beneficial effect of cyclosporine on the course of COVID-19 seems very probable. Thus, it is not justified to suggest that patients with autoimmune diseases should discontinue cyclosporine therapy solely because of the COVID-19 pandemic. However, large-scale epidemiological studies are needed to provide evidence-based medical data with clear clinical implications. 

## Figures and Tables

**Table 1:** With very limited data available, there are results that allow us to hypothesize the possible impact of cyclosporine on the course of viral diseases in humans.

Possible Positive Effect of Cyclosporine on Disease Course	Conflicting Results	Possible Negative Effect of Cyclosporine on Disease Course
Hepatitis C [15,16,17] Influenza virus infection [28] Rotavirus infection [29,30] Human Immunodeficiency Virus infection [26,33,34] Coronavirus infection [39,40,41,42,43]	Hepatitis B [10,11,12,13,14] Hepatitis D [12,20] Herpes simplex infection [26,27] Herpes Zoster Virus infection [35,36]	Hepatitis E [21] Cytomegalovirus infection [19,20,22] Human Papilloma Virus infection [29,30] Human Herpesvirus-8 (Kaposi Sarcoma virus) infection [42,43]
